# Effect of solution pH, precursor ratio, agitation and temperature on Ni-Mo and Ni-Mo-O electrodeposits from ammonium citrate baths

**DOI:** 10.3389/fchem.2022.1010325

**Published:** 2022-09-08

**Authors:** Dung T. To, Sun Hwa Park, Min Joong Kim, Hyun-Seok Cho, Nosang V. Myung

**Affiliations:** ^1^ Department of Chemical and Biomolecular Engineering, University of Notre Dame, Notre Dame, IN, United States; ^2^ Smart Devices Team, Korea Research Institute of Standards and Science, Deajeon, South Korea; ^3^ Hydrogen Research Department, Korea Institute of Energy Research, Daejeon, South Korea

**Keywords:** nickel-molybdenum alloys, nickel-molybdenum-oxide, composite, induced codeposition, hull cell, ammonium citrate baths

## Abstract

The induced co-electrodeposition of Ni and Mo is a complex process, where metallic Ni-Mo alloys and Ni-Mo-O composites can originate from the complete and partial reduction of Mo respectively. By adjusting electrolyte compositions and electrodeposition parameters, various metallic, metal/oxide composite, and oxide thin films of Ni-Mo and Ni-Mo-O were electrodeposited from ammonium citrate baths. Ni-ammonia complexes, which play a critical role in promoting the deposition of metallic Ni-Mo alloys, were enhanced at alkaline pH (*i.e.,* 8–10) and lower temperature (*i.e.*, 25–45°C). Moreover, the electrochemical reduction of Ni is under mass transfer limitation, so the deposited Mo content decreased with increasing agitation. On the other hand, higher Mo content can be achieved by relatively higher citrate concentration and larger Mo-to-Ni precursor molar ratio. However, a critical molar ratio of metal precursor resulted in transition from alloy to composite due to Ni inducing the reduction of Mo.

## 1 Introduction

Ni-Mo alloys have been employed for multiple applications due to their excellent corrosion resistance, mechanical strength, and catalytic activities (Yamada et al., 2009; Tasic et al., 2011; Mousavi et al., 2016; Abuin et al., 2019; Yuan et al., 2021). For different applications, it is essential to tune their properties by controlling their composition, morphology, and crystal structure. Electrodeposition is a powerful process technique to deposit thin films owing to its ability to tune the deposit’s properties as well as simplicity and near ambient operating conditions. Ni-Mo alloys are electrochemically reduced simultaneously through an induced co-deposition process where the reduction of Mo is catalyzed by Ni (Brenner, 1963). While the regular electrodeposition only has the electrochemical reduction at electrode-electrolyte interface, the co-deposition requires the complexation between metal precursors and complexing agents in addition to the electrochemical reduction control the relative reduction rates of both metals. Besides controlling the deposits’ composition, morphology, and crystal structure, complexing agents affect the deposit phases (i.e., metallic Ni-Mo alloys and Ni-Mo-O composite) since multi-valent Mo can be partially reduced to form oxide rather than metallic Mo. Moreover, Induced co-deposition is also influenced by both kinetics and mass transport via different synthesis parameters (e.g., solution composition, pH, current density/applied potential, and agitation) (Podlaha and Landolt, 1996). Each component *via* complexation determines the relative concentration of chemical active species for the electrochemical reduction reactions. Therefore, the composition and phase of deposits, either metallic or composite, are controlled.

Since the induced co-deposition was first reported by Brenner in 1963, many studies on the electrodeposition of Ni-Mo alloys have been achieved. However, the scope of papers were on either the complexation or the electrochemical reduction of metals (Murase et al., 2004; Hamada et al., 2008; Ohgai et al., 2013; Aaboubi et al., 2015; Péter et al., 2021). Moreover, individual papers investigated a few synthesis parameters in a narrow range, and there is no baseline condition among them (Beltowska-Lehman et al., 2012; Ohgai et al., 2013; Aaboubi et al., 2015; Bigos et al., 2017; Stryuchkova et al., 2017). Although two mechanisms were proposed for the induced co-deposition, the reports only mentioned either one (Ernst and Holt, 1958; Fukushima et al., 1979; Creager et al., 1982; Chassaing et al., 1989; Crousier et al., 1992; Rengakuji et al., 1994; Podlaha and Landolt, 1997). Therefore, a series of work which correlates the initial electrolyte composition with concentration of chemically active complexes and deposit composition to deconvolute the mechanism as well as to study the effects of synthesis parameters was conducted. In the first part of this series, the effects of ammonium-to-citrate molar ratio on the electrodeposition of Ni-Mo and Ni-Mo-O was investigated experimentally as well as correlated with the simulated fraction of species (To et al., 2022). It was suggested that ammonium ions play an important role in the fraction of chemically active nickel species and thereby the formation of metallic Ni-Mo. Additionally, most of the previous studies employed ammonium hydroxide as a pH corrector and discussed the formation of metallic alloys (Ernst and Holt, 1958; Cherkaoui et al., 1988; Chassaing et al., 1989; Beltowska-Lehman et al., 2012). In this paper, electrolyte composition (i.e., citrate concentration, molar ratio of Mo and Ni precursors, pH), operating conditions (i.e., solution temperature and agitation) and applied current density were systematically investigated. Furthermore, linear sweep voltammetry was correlated with the composition of deposits from Hull cells to investigate the deposition mechanism of Ni-Mo alloys and Ni-Mo-O composite thin films.

## 2 Experimental section

Eight different electrolytes as listed in Table 1 were investigated in this work. The concentration of Ni^2+^ and NH_4_^+^ was fixed at 0.1 M and 0.2 M respectively, other solution parameters were varied. These electrolytes were prepared by dissolving the metal precursors and the complexing agents (Podlaha and Landolt, 1996) in the sequence of sodium citrate dihydrate (ACS reagent, Sigma Aldrich), nickel sulfate hexahydrate (Certified ACS, Fisher Scientific), sodium molybdate dihydrate (ACS reagent, Sigma Aldrich), and ammonium hydroxide (ACS reagent, Sigma Aldrich) to investigate the solution composition effect. Also, the solution pH and temperature were maintained at 10 and 25°C, respectively, when they were not the studied variables. The selection of conditions is rationalized later in the results and discussion. The solution pH was adjusted using NaOH and H_2_SO_4_. Gold-coated Cu disk electrodes with a diameter of 0.1 cm were used as the working electrodes in linear sweep voltammetry whereas brass plate (267 ml brass cathode, Kocour) was used as the working electrode for Hull cell. Before electrodeposition, the substrate was cleaned with 1 M H_2_SO_4_, rinsed with deionized water, and blow dried with nitrogen gas.

**TABLE 1:** Experimental parameters for deposition of Ni-Mo alloys and Ni-Mo-O composites at fixed 0.1 M Ni^2+^ and 0.2 M NH_4._^+^

Solution #	Studied effects	Na_2_MoO_4_ (M)	Na_3_C_6_H_5_O_7_ (M)	pH	Temp. (^°^C)
1	Citrate concentration	0.05	0.05	10	25
2			0.25		
1	Mo:Ni molar ratio	0.05	0.05	10	25
3		0.1			
4		0.35			
5	Solution pH	0.05	0.05	4	25
6				5	
7				6	
8				8	
1				10	
2	Operating temp.	0.05	0.25	10	25
					45
					60
					77

The linear sweep voltammetry (LSV) was conducted using the three- electrode system (i.e., an Ag/AgCl (4 M KCl saturated with AgCl) and a Pt-coated titanium plate as the reference and counter electrodes, respectively) using a potentio-/galvano-stat (Princeton Applications VMP2) as shown in Supplementary Figure S1. Potential was swept from open circuit potential (OCP) to −2.00 V with respect to the reference electrode at the scan rate of 5 mV/s. For the Hull cell experiment, platinum coated titanium mesh was used as anode where direct current of 4A was applied by direct current (DC) power supply. To investigate the agitation effect, the paddle agitator (Kocour model A83) with a fixed rate (i.e., 7 cm/s) was employed.

The morphology of the Ni-Mo and Ni-Mo-O thin films were observed by scanning electron microscope (ThermoFisher Scientific Prisma E SEM). Energy dispersive X-ray spectroscopy (EDS) was used to characterize the composition of the thin films. The crystal structure of the films was examined by powder X-ray diffraction (XRD, Rigaku MiniFlex 600) with copper (λ = 1.5405 Å) as anticathode and 0.01-degree increments from 30 to 80ᵒ. The Gaussian fitting algorithm was utilized for the deconvolution of mixed phase peaks. The average grain size was estimated using the Scherrer equation where D_hlk_ is the crystalline size, the constant K is 0.9, B_hlk_ is the full width at half-maximum, and θ is the Bragg angle.

$$D_{hlk}=\frac{K\lambda }{B_{hlk}\cos\theta }$$ (1)

## 3 Results and discussion

### 3.1 Effects of citrate concentration

**FIGURE 1:**
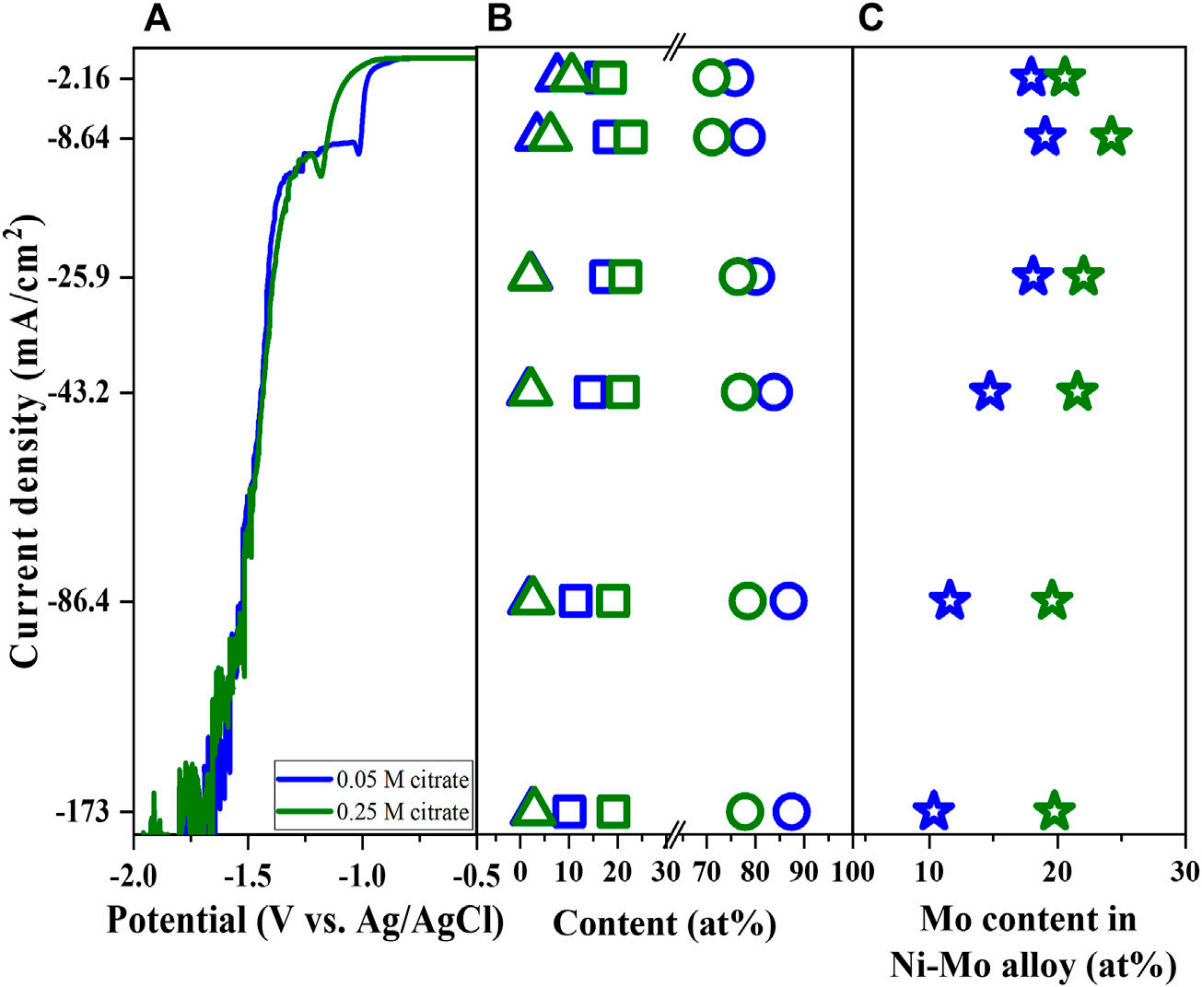
Linear sweep voltammetry **(A)**, Ni content (open circle), Mo content (open square), and O content (open triangle) of deposits **(B)**, Mo content in Ni-Mo **(C)** as a function of citrate concentration and current density at fixed 0.1 M Ni^2+^, 0.05 M MoO_4_^2−^, and 0.2 M NH_4_^+^.

In previous work, the effects of complexing agents (i.e., citrate ions and ammonia) were systematically investigated and found the key role of complexing agents in the phase transition between metallic Ni-Mo alloys and Ni-Mo-O composites as well as Ni-ammonia complexes for Ni reduction (To et al., 2022). In this work, the citrate concentration varied from 0.05 M to 0.25 M while fixing the concentration of the other components (Solutions #1 and #2) to study the effect of citrate ions on the Mo content of metallic Ni-Mo alloys. The increase of citrate concentration resulted in the polarization curve shifting to more negative values, but the magnitude of the limited current did not vary significantly. The composition of the deposits was converted to Ni-Mo alloys in Figure 1C assuming that the O content is attributed to surface oxidation after deposition. The highest Mo content in Ni-Mo alloys for both conditions occurred at the similar current density which might be attributed to the reduction of chemically active Ni-ammonia complexes. The complete reduction of MoO_4_^−^ to metallic Mo (0) is determined by the availability of Ni atoms and adsorbed H atoms, which explains the highest Mo content at the diffusion limiting reduction of Ni. In addition, a high rate of hydrogen evolution reaction (HER) at more negative applied potential might enhance the mass transport of Ni and Mo species by the rising bubbles of H_2_ gas (Podlaha et al., 1993). The highest Mo content (i.e., 24.2 at% Mo) in the alloys was observed at the limited current density and reduced at higher applied potentials, indicating the greater effect of hydrogen gas evolution on Ni species than Mo species. For electrodeposition of Ni-Mo in citrate ammonium bath, Ernst and Holt (1958) reported the reduction of Mo content in the deposits with increase in current density. This was suggested to be attributed to the higher activation-limited reduction of Ni-citrate species and lower diffusion-limited reduction of Mo species (Cherkaoui et al., 1988; Chassaing et al., 1989; Beltowska-Lehman et al., 2012). As shown in Figure 1B, the lower deposited Ni content at higher initial concentration of citrate (e.g., more Ni-citrate complexes and less Ni-ammonia complexes) indicates that the higher Ni reduction at higher current density probably comes from the effect of H_2_ bubbling or mass transport on reduction of Ni-ammonia species.

The higher initial concentration of citrate resulted in a lower deposited Ni content, implying the Ni-citrate complexes are not favorable for Ni reduction. More citrate ions in electrolyte leads to greater Ni-citrate complexes compared to Ni-ammonia complexes. The effect of citrate concentration further confirms that Ni-ammonia complexes are presumably the chemically active species for Ni reduction, particularly Ni(NH_3_)_3_^2+^ (To et al., 2022). Since Ni and Mo content in metallic Ni-Mo alloys are inversely proportional, the higher Mo content might be owing to lower relative Ni content. This is further confirmed by the oxygen content in the similar range (Figure 1B). Increasing the citrate concentration in the presence of ammonium was reported to improve the Mo content but a limit value was found not to further increase the Mo content (Podlaha and Landolt, 1996).

**FIGURE 2:**
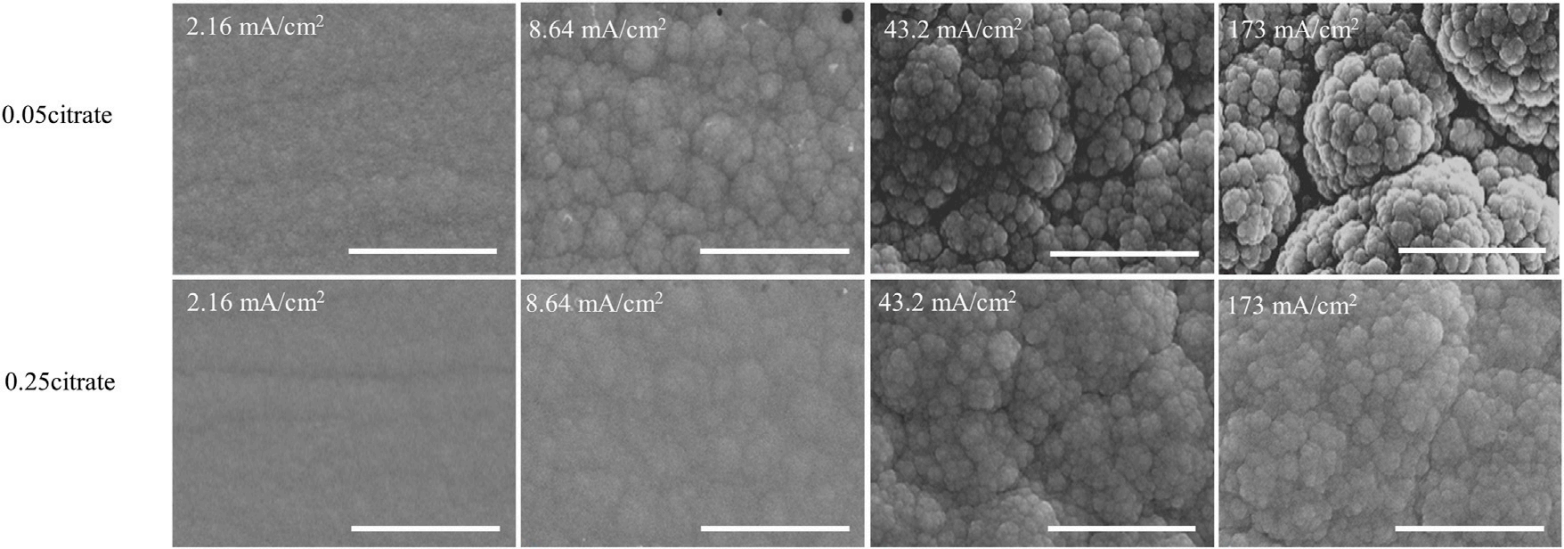
SEM images of deposit as a function of citrate concentration and current density at fixed 0.1M Ni^2+^, 0.05M MoO_4_^2−^, and 0.2 M NH_4_^+^. Scale bar: 3 µm.

Morphology of the electrodeposits was characterized by scanning electron microscopy (SEM) at four current densities (i.e., 2.16, 8.64, 43.2, and 173 mA cm^−2^). All conditions resulted in the typical nodular structure of metallic Ni-Mo alloys as shown in Figure 2. The transition to more roughly nodular structure was also observed with increase in current densities. Three samples at current densities of 2.16, 8.64, and 173 mA/cm^2^ from solution #2 were selected for the XRD characterization. The calculated average grain size for the (111) peak of face-centered cubic phase (JCPDS file #04–0850) ranges from 3.99 to 3.40 nm, indicating the deposits are nanocrystalline with similar grain size. Furthermore, the XRD patterns suggest that the Mo content has more effect on the crystal structure of deposit than the initial citrate concentration. The spectra of three samples were selectively shown in Supplementary Figure S2. Increasing current density from 8.16 to 173 mA/cm^2^, the Mo content of deposits from solution #1 changes from 19.0 to 10.4 at%, corresponding to the transition from orthorhombic phase (JCPDS file #47–1129) to face-centered cubic phase (JCPDS file #04–0850). Comparing the standard and the spectrum of 10.4 at% Mo, a peak shift to lower 2θ values was also observed probably due to the incorporation of larger Mo atoms into the Ni lattice. On the other hand, the metallic Ni-Mo alloys with about 20 at% Mo from solution #1 at 8.64 mA/cm^2^ and solution #2 at 173 mA/cm^2^ have the orthorhombic phase.

### 3.2 Effects of Mo/Ni precursor molar ratio

**FIGURE 3:**
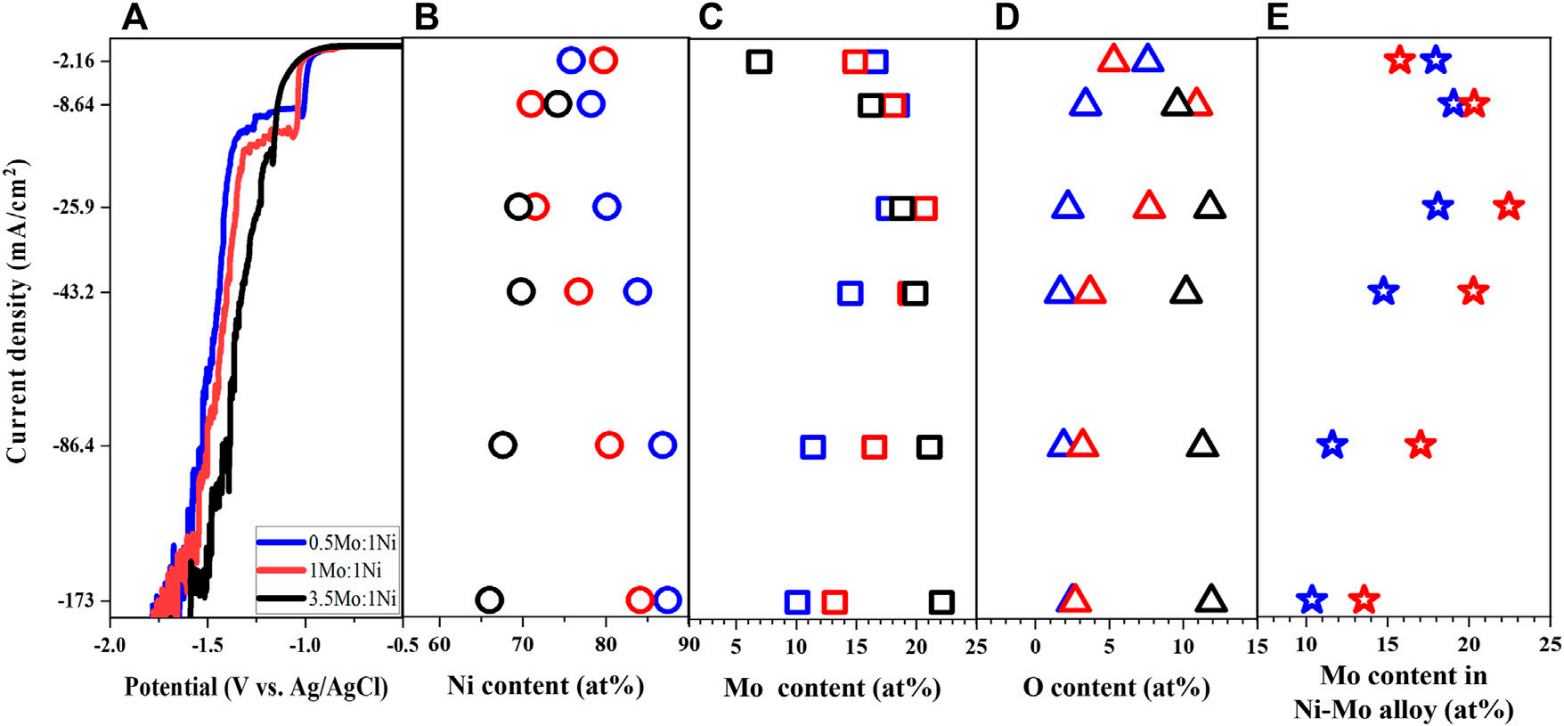
Linear sweep voltammetry **(A)**, Ni content **(B)**, Mo content **(C)**, O content **(D)**, and Mo content in Ni-Mo alloys **(E)** of deposits as a function of Mo-to-Ni molar ratio at fixed 0.1 M Ni^2+^, 0.05 M Cit^3−^, and 0.2 M NH_4_^+^.

Besides complexing agents, the Ni-to-Mo precursor molar ratio is another important parameter which influences the reaction kinetics. While the concentration of nickel, citrate and ammonium ions were fixed as shown in Table 1, the Mo precursor concentration was controlled to vary the Mo-to-Ni molar ratio from 0.5 to 3.5 (Solutions # 1, 3, 4) to study both Ni-rich and Mo-rich solutions. The shift of LSV in Figure 3A to more negative values at the lower range of applied potential suggests that reduction of Ni and Mo require more energy as the metal precursor molar ratio increases. The higher concentration of Mo precursor leads to the limit current over a shorter range of potential and the reverse trend of the polarization curves at the higher applied potential.

As expected, the deposited Mo content increased with increasing the concentration of Mo precursor (Higashi et al., 1976; Fukushima et al., 1979; Rengakuji et al., 1995); however, the highest Mo-to-Ni molar ratio resulted in the formation of Ni-Mo-O composite instead of metallic Ni-Mo as shown in Figures 3B–D. The phase transition might be attributed to the lower relative ratio of the reduced Ni content and MoO_4_^2−^ ions, which led to partial reduction of MoO_4_^2−^ ions. Furthermore, it is interesting to observe the consistency of the trend between the limited current and the highest Mo content with respect to the current density. The larger limited currents from higher concentration of Mo precursor resulted in higher Mo content in the Ni-Mo metallic alloys. The effect of current density on the composition of the Ni-Mo alloys in Figure 3E agrees with that in Figure 1C because of the HER.

**FIGURE 4:**
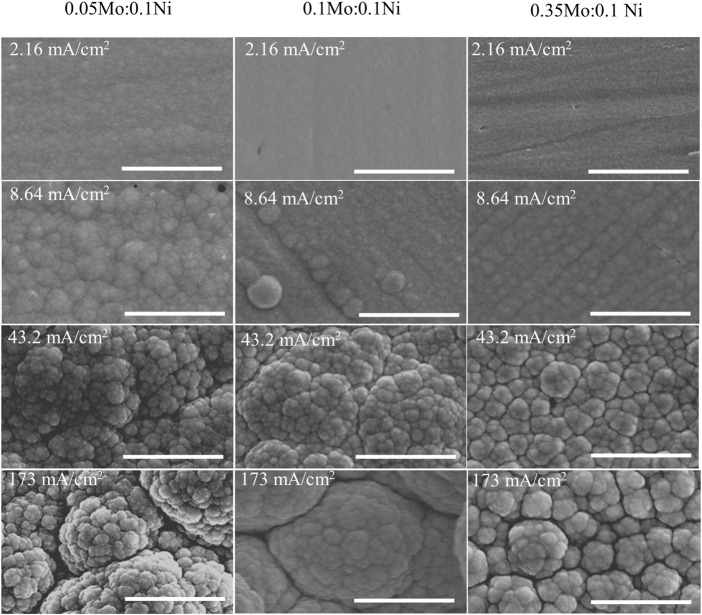
SEM images of deposits at various Mo-to-Ni molar ratio and current density at fixed 0.1 M Ni^2+^, 0.05 M Cit^3−^, and 0.2 M NH_4_^+.^ Scale bar: 3 µm.

As shown in Figure 4, the morphology of the deposits shows a similar transition as observed from the effects of ammonium-to-citrate molar ratio. The surface became more globular at higher current density, which was independent of the electrolyte composition. In addition, the higher oxygen content or the less metallic deposit is associated with smoother surface or smaller globular structures. The XRD data (not shown) of samples at 25.9 mA/cm^2^ is consistent with the roughness in which larger Mo precursor concentrations resulted in smaller grain size in the range of 1.29 nm and 4.31 nm. Although three samples have the orthorhombic phase, the larger ratio of Mo-to-Ni precursor concentration leads to broader peaks or more amorphous deposits.

### 3.3 Solution pH effect

**FIGURE 5:**
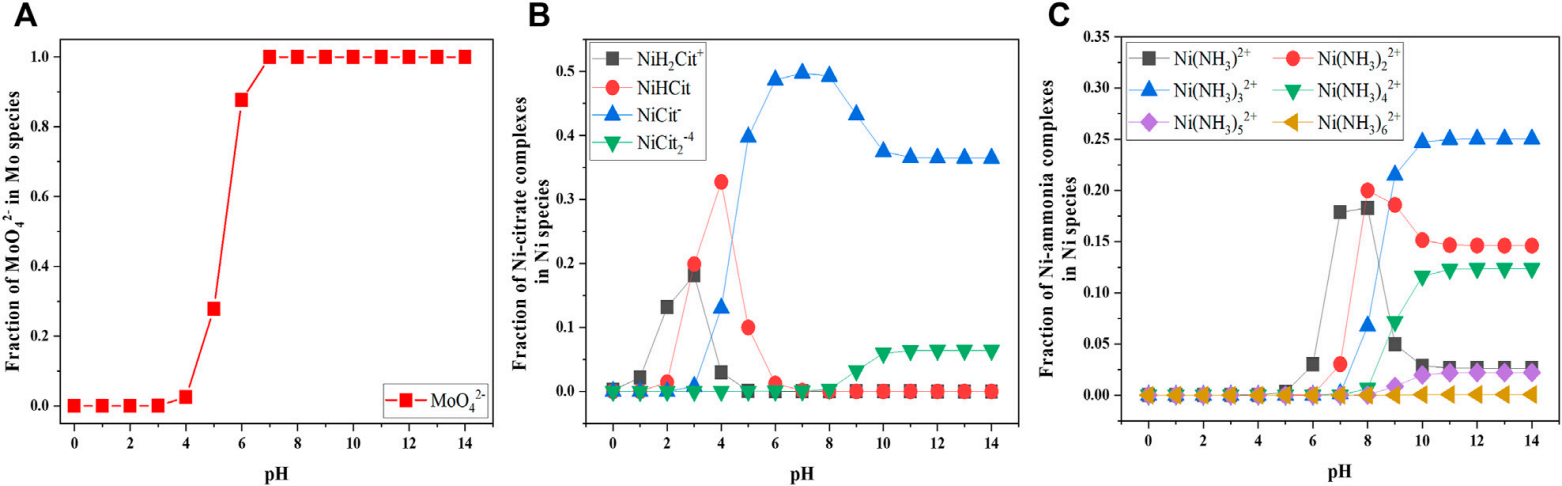
Simulated fraction of MoO_4_^2−^ **(A)**, Ni citrate complexes **(B)**, and Ni ammonia complexes **(C)** as a function of pH concentration at fixed 0.1 M Ni^2+^, 0.05 M MoO_4_^2−^, 0.05 M Cit^3−^.

Owing to the pH-dependent fraction of Ni and Mo species, solution pH is a crucial factor which determines the chemically active species for Ni reduction as well as the relative concentration of MoO_4_^2−^ and the active Ni species. Utilizing the commercial programming and numeric computing platform (i.e., MATLAB from Mathwork^®^) to simulate the fraction of species as shown in Figure 5, five pH conditions (i.e., 4–6, 8 and 10) were selected to study a wide range of fraction of MoO_4_^2−^ ions. Moreover, the lower pH range was designated for the effect of NiCit^−^ due to the negligible fraction of Ni(NH_3_)_3_^2+^. In contrast, the alkaline pH at 8 and 10, where different fractions of Ni(NH_3_)_3_^2+^ exist, were chosen to investigate the effect of Ni-ammonia complexes.

**FIGURE 6:**
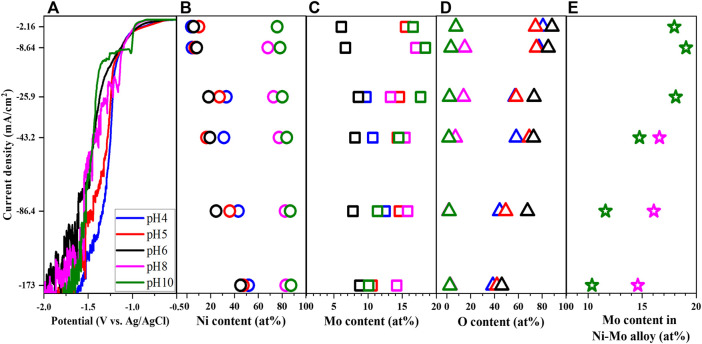
Linear sweep voltammetry **(A)**, Ni content **(B)**, Mo content **(C)**, O content **(D)** and Mo content in Ni-Mo alloy **(E)** of deposits as a function of pH at fixed 0.1 M Ni^2+^, 0.05 M MoO_4_^2−^, 0.05 M Cit^3−^, and 0.2 M NH^4+^.

In comparison to the previously discussed parameters, the trend of LSV becomes more diverse with varied pH as observed in Figure 6A which might be attributed to the variation of all chemically active species for the reduction of Ni and Mo. The polarization curves at the lower range of applied potential generally have two shapes with the reduction peak and the limited current at the higher pH range and without them at the lower pH range. Provided the understanding about the variation of polarization curves from other parameters, the phases of the deposits can be predicted to be Ni-Mo alloys at pH 8 and 10 and Ni-Mo-O composites at pH 4-6 correspondingly. This is consistent with the analysis of deposit composition in Figures 6B–D. Moreover, the lower pH shifts the polarization curves to more positive potential, attributed to lower overpotential of HER at more acidic pH. Chassaing et al. (2004) suggested the more significant hydrogen gas evolution at lower pH resulted in the shift of cathodic polarization curve toward more negative potentials when the deposition occurred in more alkaline solutions.

Besides, the higher concentration of Ni(NH_3_)_3_^2+^ at pH 10 resulted in lower O content, higher Ni content and thereby lower Mo content in the Ni-Mo alloys, compared to the deposit composition at pH 8. Correlating the fraction of species in Figure 5 and the data in Figure 6, the presence of Ni(NH_3_)_3_^2+^ as well as the relative concentration of Ni(NH_3_)_3_^2+^ and NiCit^−^ might play a crucial role in the formation of metallic and oxides phases and tunability of Mo content. As indicated in Figure 6E, the lower concentration of Ni(NH_3_)_3_^2+^ at pH 8 requires higher applied potential for sufficiently reduced Ni atoms and thereby the complete reduction of MoO_4_^2−^ to form metallic alloys.

Compared to the other conditions resulting in the formation of metallic Ni-Mo alloys, the morphology of deposits at pH 8 and 10 had the nodular structure and the surface roughness grew with increase in current density. At the lower pH range, there was the formation of microcracks within the deposits as shown in Supplementary Figure S3. Also, the deposits at pH 4 grew nodular structures whose density reduced at higher pH which might be associated with the higher O content or less metallic deposits. The deposits at 173 mA/cm^2^ were chosen for XRD characterization and their spectra were plotted in Supplementary Figure S4. Consistent with the composition and morphology, the samples from higher pH range (i.e., 8 and 10) have the fcc phase with peak shift to lower angle of 2θ. In addition, the pH 10 solution results in the metallic Ni-Mo alloys with larger grain size than the pH 8 solution (i.e., 5.78 nm vs. 2.86 nm). In contrast, the deposits from lower pH solutions have an orthorhombic phase with the grain size less than 2.37 nm. The peaks become broadened with higher pH, and pH 6 leads to formation of amorphous film with no observable peaks. Correlating with composition, the electrodeposits from lower pH solution might consist of the orthorhombic phase of Ni-Mo in the matrix of amorphous oxide.

### 3.4 Effects of reaction temperature

**FIGURE 7:**
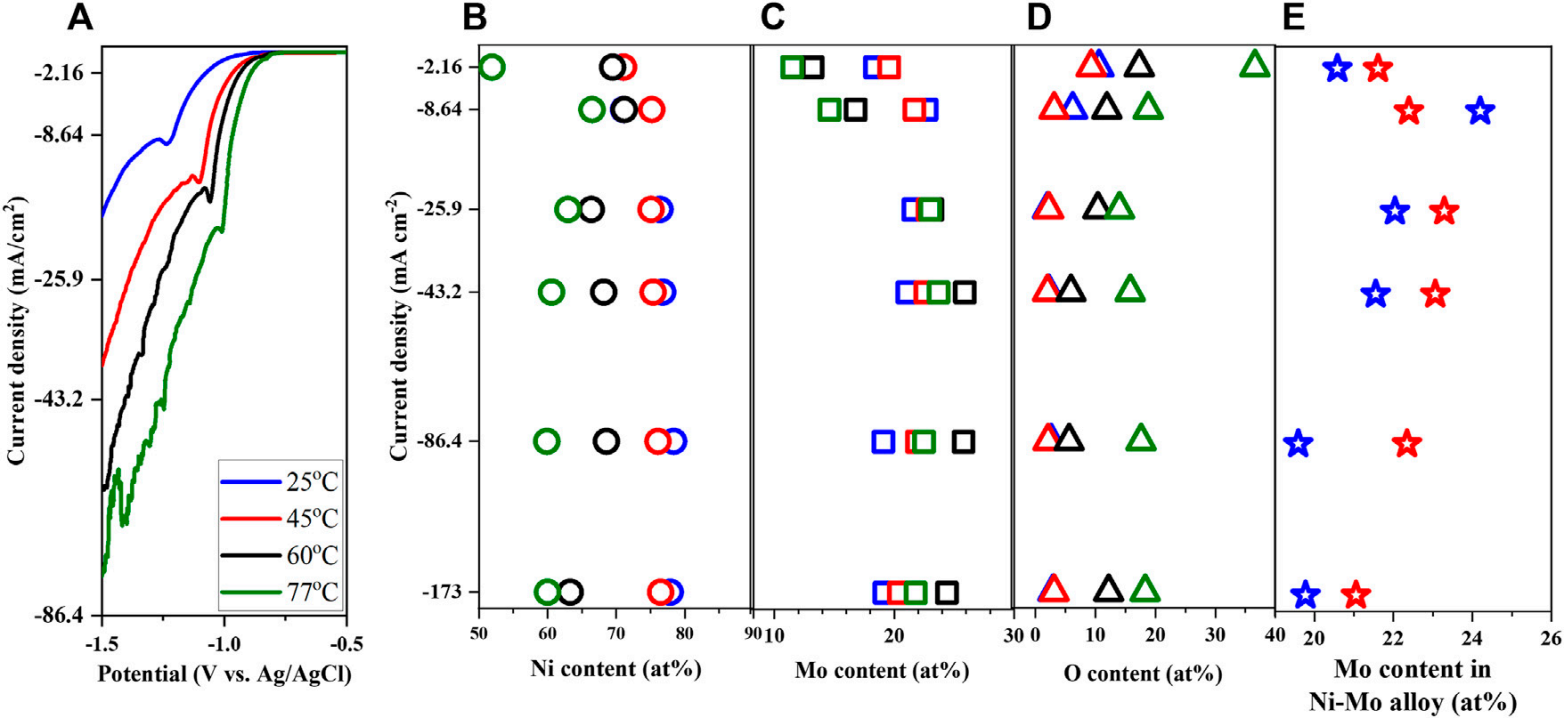
Linear sweep voltammetry **(A)**, Ni content **(B)**, Mo content **(C)**, O content **(D)** and Mo content in Ni-Mo alloy **(E)** of deposits as a function of solution temperature at fixed 0.1 M Ni^2+^, 0.05 M MoO_4_^2−^, 0.25 M Cit^3−^, and 0.2 M NH^4+^.

Reaction temperature which controls both thermodynamics and kinetics of a reaction is a very important synthesis parameter to study. As the deposited alloys possess the highest Mo content from the study of citrate concentration effect, the citrate concentration employed for the effect of solution temperature is 0.25 M. The solution pH and concentration of nickel, molybdate, and ammonium ions were fixed at 10, 0.1 M, 0.05 M, and 0.2 M respectively (Solution # 2). The temperature range between 25°C and 77°C was chosen not only to maximize the range but also to avoid the interference of water evaporation. Increment of temperature moved the LSV curves to more positive values as displayed in Figure 7A, implying the smaller required energy for the reduction of Ni and Mo. This might be due to the higher diffusivity of ions, thereby higher reactant concentrations in the diffusion layer at higher temperatures. Mousavi et al. (2016) reported the shift of polarization curves to more positive potentials as temperature increased from 25 to 40°C at pH 9 using citrate ammonium bath. However, the composition analysis in Figures 7B–D suggests the phase transition from metallic to composite deposits at higher temperature. This discrepancy might be attributed to the relative short reaction time of LSV (i.e.*,* 5 min) in comparison to that of Hull cell (i.e., 20 min). At higher temperature, the vapor pressure of ammonia becomes larger, facilitating the evaporation of ammonia and consequently reducing the concentration of Ni-ammonia complexes in the electrolyte. In other words, increasing the solution temperature might promote the reduction of Ni and Mo but also possibly depresses it by lowering the concentration of the chemically active species for Ni reduction. Looking at the composition of the metallic deposits in Figures 7B,C, the reduction of Ni content and augmentation of Mo might be attributed to lower concentration of the Ni-ammonia complex and larger concentration of MoO_4_^2−^ as the solution temperature increased from 25°C to 45°C. Studying the temperature effect between 25°C and 40°C, Bigos et al. reported that temperature above 40°C ceased the stability of plating solution (Bigos et al., 2017). In accordance with the observation of Podlaha and Landolt (1996), the maximum Mo content occurred at the higher current density with increase in solution temperature as shown in Figure 7E.

The morphology transitions from globular structure to smooth surface in Supplementary Figure S5 is probably owing to the phase transition from metallic to composite deposits as the solution temperature increases. Additionally, the surface roughness becomes more pronounced at higher current density as expected. Characterized samples at 173 mA/cm^2^ exhibit similar XRD patterns (data not shown) with the grain size below 3.04 nm. Moreover, higher solution temperatures cause the broadened peaks, implying the deposits become more amorphous as transitioning from metallic to composite phases.

### 3.5 Effects of agitation

**FIGURE 8:**
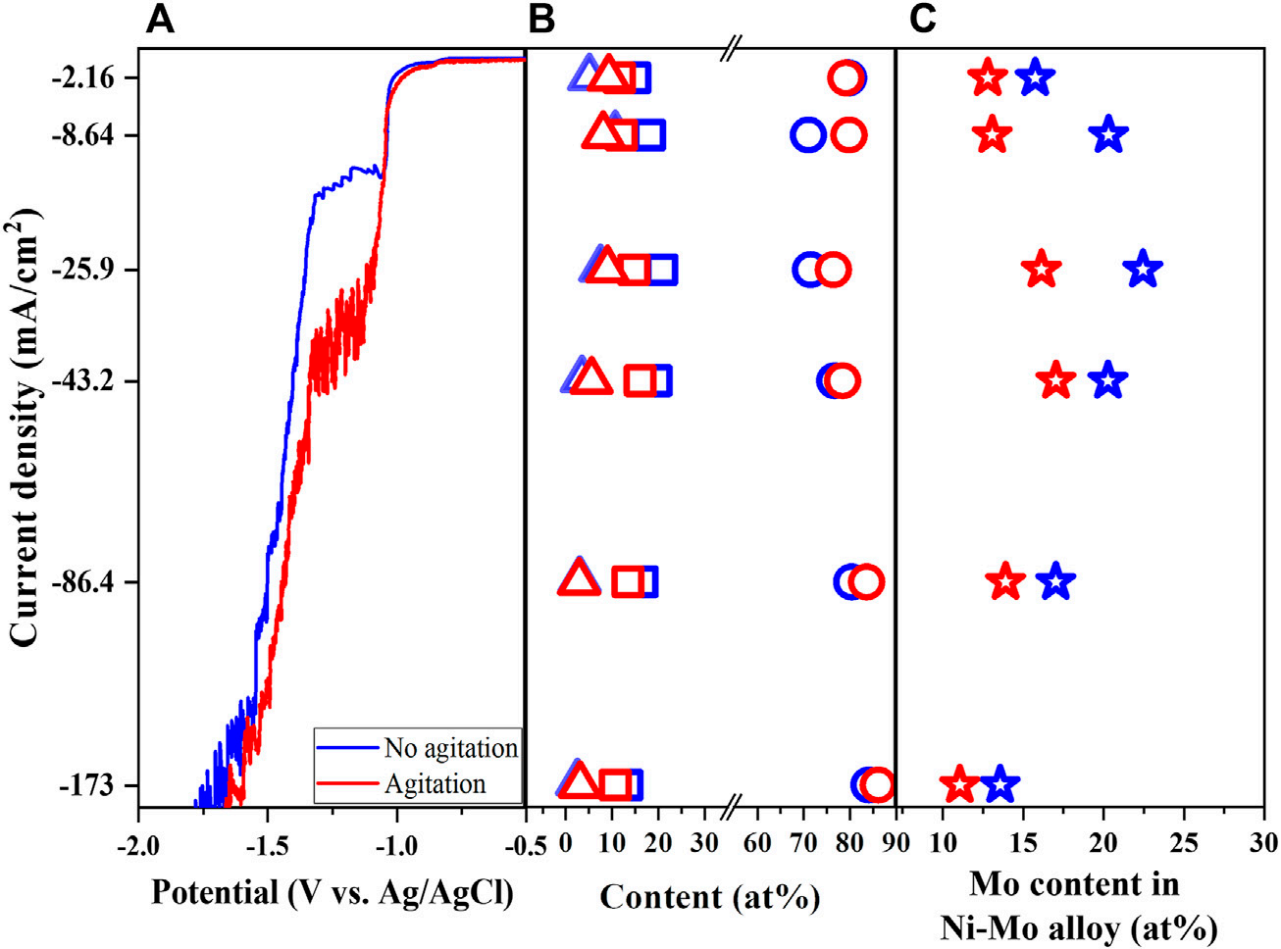
Linear sweep voltammetry **(A)**, Ni content (open circle), Mo content (open square), and O content (open triangle) of deposits **(B)**, Mo content in Ni-Mo **(C)** as a function of agitation and current density at fixed 0.1 M Ni^2+^, 0.1 M MoO_4_^2−^, 0.05 M Cit^3−^ and 0.2 M NH_4_^+^.

To study the effect of mass transport on the formation and composition of Ni-Mo alloys, the paddle agitation was introduced to the solution consisting of 0.1 M of Ni and Mo precursors, 0.05 M citrate, 0.2 M ammonium and pH 10 at 25°C (Solution #3). The effect of agitation on the LSV becomes pronounced at the larger range of applied potential in which the polarization curve shifts to a more positive value, implying less energy required for the reduction under stirring. The agitation also increased the limited current, indicating the enhanced mass transport of both Ni and Mo species. The shift corresponds to the exhibition of the highest Mo content at a higher current density as shown in Figure 8C. The composition of deposits in Figure 8B suggests the enhancement of Ni reduction and/or decrease of Mo reduction with agitation. Consequently, the smaller Mo content in Ni-Mo alloys is observed in Figure 8C might be due to the larger effect of agitation on the transport of Ni species than that of Mo species at the equal Ni and Mo precursor concentrations. Podlaha and Landolt (1996) reported that the Mo reduction rate was limited by the mass transport of molybdenum ions in the Ni-rich solution and of nickel ions in the Mo-rich solution, respectively. The Ni-rich solutions (i.e., Ni/Mo molar ratio > 4) under excess ammonium and hydrodynamic conditions resulted in higher Mo content alloys in comparison to the unagitated solution (Chassaing et al., 2004; Bigos et al., 2017). This is probably due to the activation control of Ni reduction from Ni-citrate complexes and agitation promoting only the transport-limited reduction of Mo.

As expected, the nodular structures were formed for the metallic Ni-Mo alloys and the roughness increases with higher current density as shown in Supplementary Figure S6. The nodular structures reduced in size under the influence of agitation as Bigos *et al.* also observed by using the rotating disk electrode (Bigos et al., 2017).

## 4 Conclusion

The systematic study provides the effects of various electrodeposition parameters and solution composition on the deposit phases and composition by deconvolute the deposition mechanism. Since electrochemically reduced Ni catalyzes the reduction of Mo and Ni-ammonia complexes are more favorable for Ni reduction, the high concentration of Ni-ammonia complexes facilitates the formation of metallic Ni-Mo alloys. Moreover, higher reduced Ni corresponds to lower Mo content due to their inversely proportional correlation in alloys. Within the solution conditions forming metallic alloys, increasing the citrate concentration reduces the Ni content and thereby increases the Mo content due to lower fraction of Ni-ammonia complexes. In contrast, introduction of agitation improves the mass transport of Ni precursor more than Mo precursor, leading to lower Mo content in alloys. In addition to complexing agents, Mo-to-Ni molar ratio plays a crucial role in increasing the Mo content of alloys due to mitigating the mass transport limitation and transitioning from alloys to composites due to excessive Mo precursor. The phase transition was also observed when changing pH from alkaline (*i.e.,* 8–10) to acidic (*i.e.,* 4–6) conditions or increasing temperatures.

## Data Availability

The raw data supporting the conclusions of this article will be made available by the authors, without undue reservation.

## References

[B1] Aaboubi O. Ali Omar A. Y. Franczak A. Msellak K. (2015). Investigation of the electrodeposition kinetics of Ni–Mo alloys in the presence of magnetic field. J. Electroanal. Chem. 737, 226–234. 10.1016/j.jelechem.2014.10.014

[B2] Abuin G. Coppola R. Diaz L. (2019). Ni-Mo alloy electrodeposited over Ni substrate for HER on water electrolysis. Electrocatalysis 10 (1), 17–28. 10.1007/s12678-018-0490-2

[B3] Beltowska-Lehman E. Bigos A. Indyka P. Kot M. (2012). Electrodeposition and characterisation of nanocrystalline Ni–Mo coatings. Surf. Coatings Technol. 211, 67–71. 10.1016/j.surfcoat.2011.10.011

[B4] Bigos A. Beltowska-Lehman E. Kot M. (2017). Studies on electrochemical deposition and physicochemical properties of nanocrystalline Ni‐Mo alloys. Surf. Coatings Technol. 317, 103–109. 10.1016/j.surfcoat.2017.03.036

[B5] Brenner A. (1963). Electrodeposition of alloys. New York: Academic Press.

[B6] Chassaing E. Portail N. Levy A-f. Wang G. (2004). Characterisation of electrodeposited nanocrystalline Ni–Mo alloys. J. Appl. Electrochem. 34 (11), 1085–1091. 10.1007/s10800-004-2460-z

[B7] Chassaing E. Vu Quang K. Wiart R. (1989). Mechanism of nickel-molybdenum alloy electrodeposition in citrate electrolytes. J. Appl. Electrochem. 19 (6), 839–844. 10.1007/bf01007931

[B8] Cherkaoui M. Chassaing E. Vu Quang K. (1988). Plating OF NI-mo alloy coatings. Adv. Mater. Manuf. Process. 3 (3), 407–418. 10.1080/08842588708953213

[B9] Creager S. E. Aikens D. A. Clark H. M. (1982). The electroactive Mo(VI) species in neutral citrate medium. Electrochimica Acta 27 (9), 1307–1310. 10.1016/0013-4686(82)80152-7

[B10] Crousier J. Eyraud M. Crousier J. P. Roman J. M. (1992). Influence of substrate on the electrodeposition of nickel-molybdenum alloys. J. Appl. Electrochem. 22 (8), 749–755. 10.1007/bf01027505

[B11] Ernst D. W. Holt M. L. (1958). Cathode potentials during the electrodeposition of molybdenum alloys from aqueous solutions. J. Electrochem. Soc. 105 (11), 686. 10.1149/1.2428691

[B12] Fukushima H. Akiyama T. Akagi S. Higashi K. (1979). Role of iron-group metals in the induced codeposition of molybdenum from aqueous solution. Trans. JIM. 20 (7), 358–364. 10.2320/matertrans1960.20.358

[B13] Hamada Y. Bayakly N. George D. Greer T. (2008). Speciation of molybdenum(VI)-Citric acid complexes in aqueous solutions. Synthesis React. Inorg. Metal-Organic Nano-Metal Chem. 38, 664–668. 10.1080/15533170802371323

[B14] Higashi K. Fukushima H. Ohashi H. Akiyama T. (1976). Effects of alkali cations on the electrodeposition of nickel-molybdenum alloys from the ammonical tartrate bath. J. Metal Finish. Soc. Jpn. 27 (11), 590–595. 10.4139/sfj1950.27.590

[B15] Mousavi R. Bahrololoom M. E. Deflorian F. Ecco L. (2016). Improvement of corrosion resistance of NiMo alloy coatings: Effect of heat treatment. Appl. Surf. Sci. 364, 9–14. 10.1016/j.apsusc.2015.12.041

[B16] Murase K. Ogawa M. Hirato T. Awakura Y. (2004). Design of acidic Ni-Mo alloy plating baths using a set of apparent equilibrium constants. J. Electrochem. Soc. 151 (12), C798. 10.1149/1.1817758

[B17] Ohgai T. Tanaka Y. Washio R. (2013). Nanocrystalline structure and soft magnetic properties of nickel–molybdenum alloy thin films electrodeposited from acidic and alkaline aqueous solutions. J. Solid State Electrochem. 17 (3), 743–750. 10.1007/s10008-012-1924-z

[B18] Péter L. Fekete É. Kapoor G. Gubicza J. (2021). Influence of the preparation conditions on the microstructure of electrodeposited nanocrystalline Ni–Mo alloys. Electrochimica Acta 382, 138352. 10.1016/j.electacta.2021.138352

[B19] Podlaha E. Matlosz M. Landolt D. (1993). Electrodeposition of high Mo content Ni‐Mo alloys under forced convection. J. Electrochem. Soc. 140 (10), L149–L151. 10.1149/1.2220956

[B20] Podlaha E. J. Landolt D. (1996). Induced codeposition: I. An experimental investigation of Ni‐Mo alloys. J. Electrochem. Soc. 143 (3), 885–892. 10.1149/1.1836553

[B21] Podlaha E. J. Landolt D. (1997). Induced codeposition: III. Molybdenum alloys with nickel, cobalt, and iron. J. Electrochem. Soc. 144 (5), 1672–1680. 10.1149/1.1837658

[B22] Rengakuji S. Nakamura Y. Nishibe K. Inoue M. Komura T. (1994). A mechanistic consideration on the electrodeposition of Ni-Mo alloys. Denki Kagaku1961. 62 (7), 602–606. 10.5796/electrochemistry.62.602

[B23] Rengakuji S. Nakamura Y. Sumi N. Nishibe K. Komura T. (1995). Stress in Ni-Mo alloys electrodeposited from ammoniacal citrate solution. Denki Kagaku1961. 63 (5), 400–405. 10.5796/kogyobutsurikagaku.63.400

[B24] Stryuchkova Y. M. Rybin N. B. Suvorov D. V. Gololobov G. P. Tolstoguzov A. B. Tarabrin D. Y. (2017). Study of Ni-Mo electrodeposition in direct and pulse-reverse current. J. Phys. Conf. Ser. 857, 012046. 10.1088/1742-6596/857/1/012046

[B25] Tasic G. S. Maslovara S. P. Zugic D. L. Maksic A. D. Kaninski M. P. M. (2011). Characterization of the Ni–Mo catalyst formed *in situ* during hydrogen generation from alkaline water electrolysis. Int. J. Hydrogen Energy 36 (18), 11588–11595. 10.1016/j.ijhydene.2011.06.081

[B26] To D. T. Park S. H. Kim M. Cho H.-S. Myung N. V. (2022). Effects of NH^4+^/citrate complexing agent ratio on Ni–Mo and Ni–Mo–O electrodeposits from ammonium citrate baths. Front. Chem.. 10.3389/fchem.2022.942423 PMC9468647 36110137

[B27] Yamada Y. Miyata S. Yoshizumi M. Fukushima H. Ibi A. Izumi T. (2009). Long IBAD-MgO and PLD coated conductor. Phys. C. Supercond. 469 (15), 1298–1302. 10.1016/j.physc.2009.05.137

[B28] Yuan W. Cui Z. Zhu S. Li Z. Wu S. Liang Y. (2021). Structure engineering of electrodeposited NiMo films for highly efficient and durable seawater splitting. Electrochimica Acta 365, 137366. 10.1016/j.electacta.2020.137366

