# Supplementary material for: Effect of solution pH, precursor ratio, agitation and temperature on Ni-Mo and Ni-Mo-O electrodeposits from ammonium citrate baths
Source: Front Chem. 2022 Sep 8;10:1010325. doi: 10.3389/fchem.2022.1010325 (PMC9521569; doi:10.3389/fchem.2022.1010325)
Supplement: Supplementary file 1 [file DataSheet1.docx]

**Effect of Solution pH, Precursor Ratio, Agitation and Temperature on Ni-Mo and Ni-Mo-O Electrodeposits from Ammonium Citrate Baths**

**
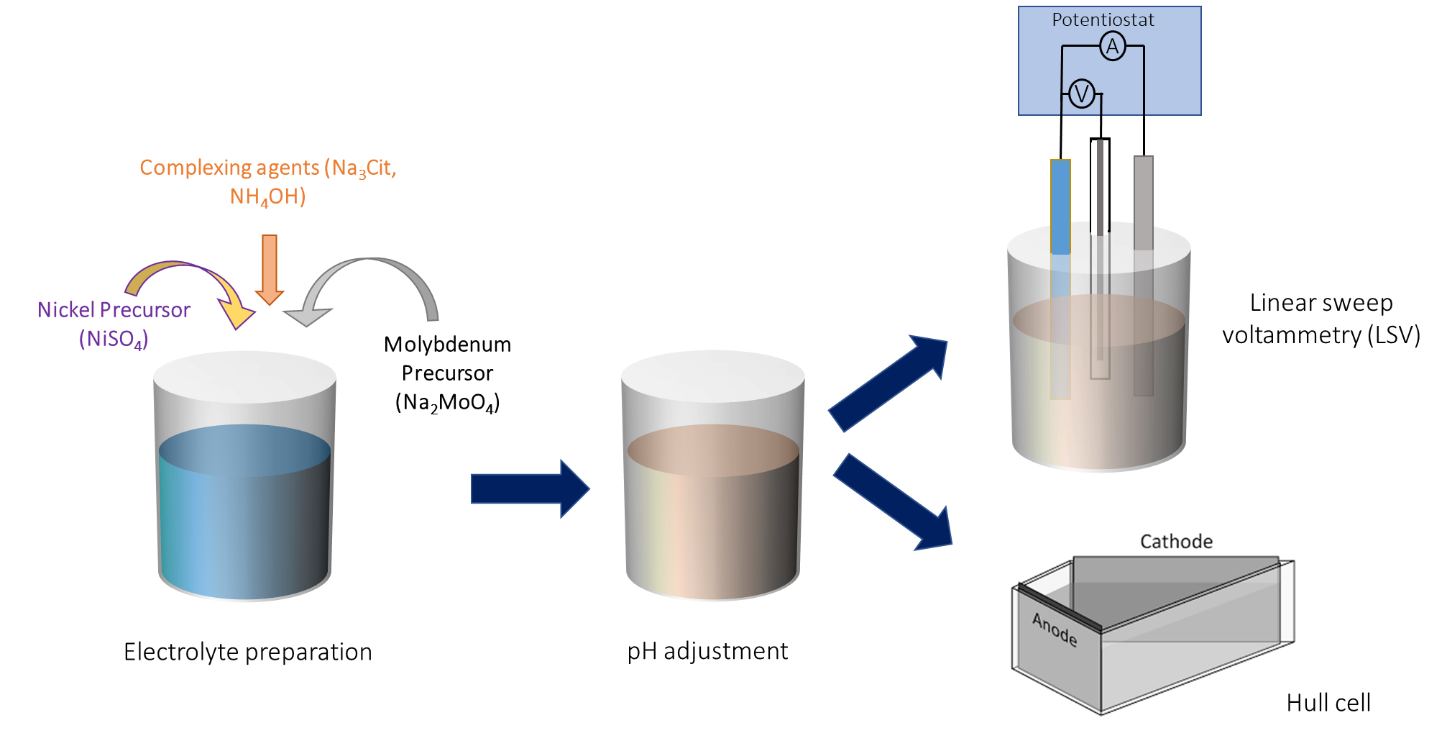
**

**Figure S1.** Schematic diagram of synthesis of Ni-Mo and Ni-Mo-O electrodeposits

**Figure S2**. XRD spectra of samples at different citrate concentration (solutions #1 and #2) and fixed 0.1 M Ni^2+^, 0.05 M MoO_4_^2-^, 0.05 M Cit^3-^, and 0.2 M NH_4_^+^. S represents substrate.


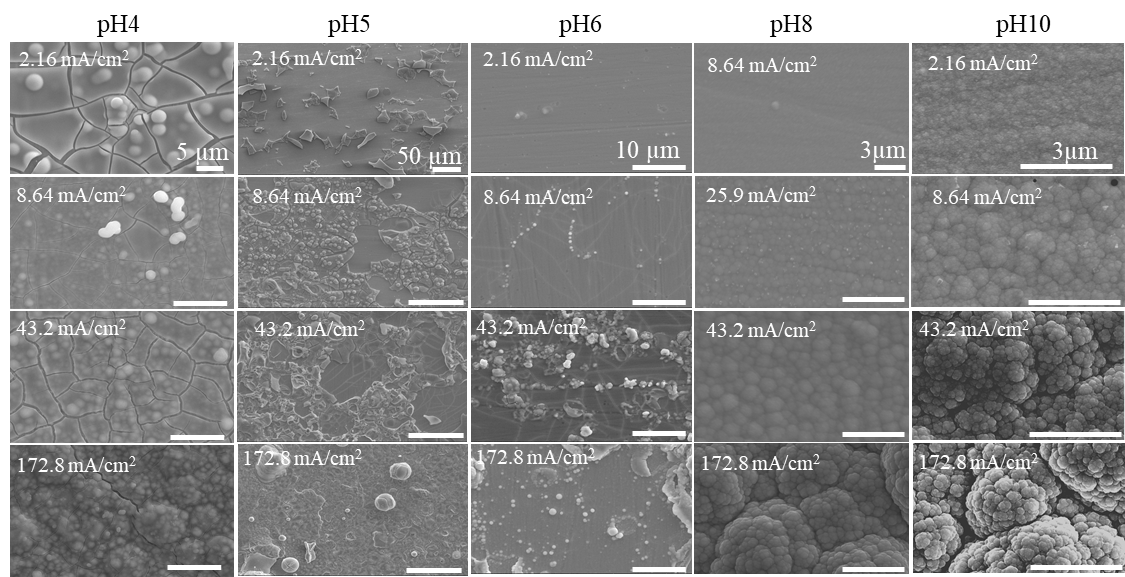


**Figure S3**: SEM images of deposits at various pH and current density at fixed 0.1 M Ni^2+^, 0.05 M MoO_4_^2-^, 0.05 M Cit^3-^, and 0.2 M NH_4_^+^. Scale bar: 3 µm

**Figure S4.** XRD spectra of samples at 173 mA/cm2 at different pH (solutions #1 and #5-8) and fixed 0.1 M Ni^2+^, 0.05 M MoO_4_^2-^, 0.05 M Cit^3-^, and 0.2 M NH_4_^+^. S represents substrate.

45^o^C

77^o^C

60^o^C

25^o^C


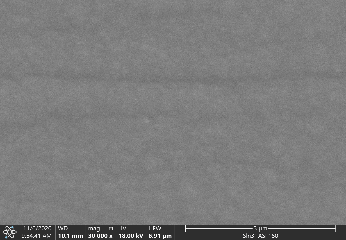


2.16 mA/cm^2^


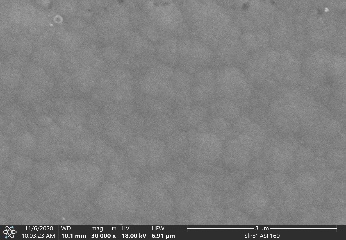


8.64 mA/cm^2^


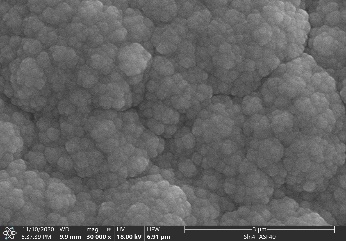


43.2 mA/cm^2^


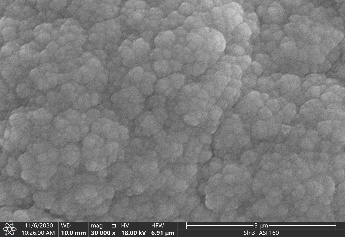


172.8 mA/cm^2^


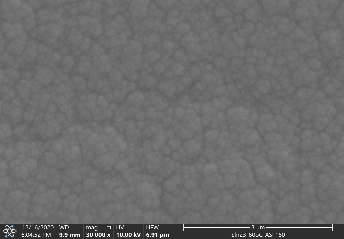

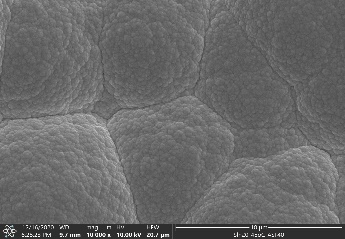


172.8 mA/cm^2^

172.8 mA/cm^2^


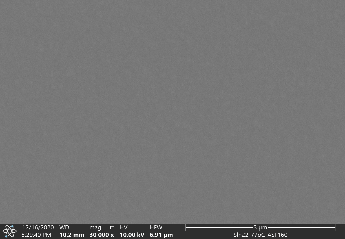


172.8 mA/cm^2^


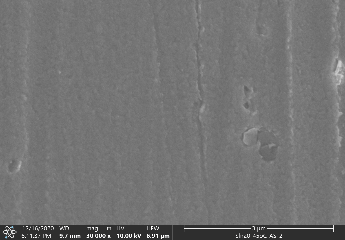


2.16 mA/cm^2^


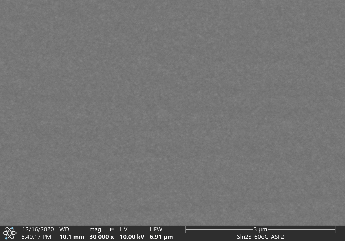


2.16 mA/cm^2^


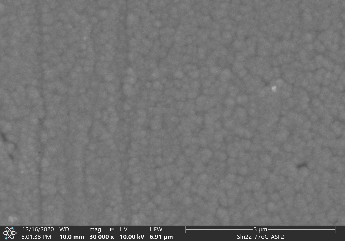


2.16 mA/cm^2^


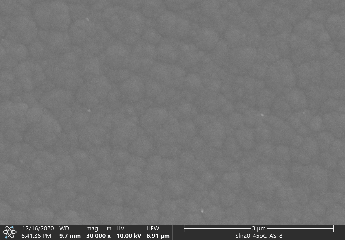


8.64 mA/cm^2^


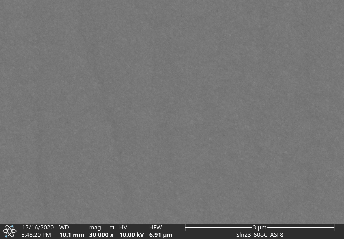


8.64 mA/cm^2^


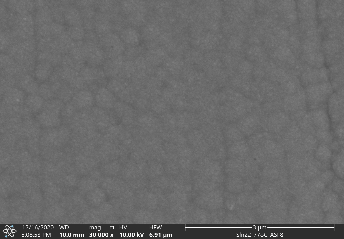


8.64 mA/cm^2^


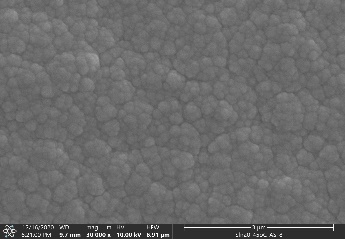


43.2 mA/cm^2^


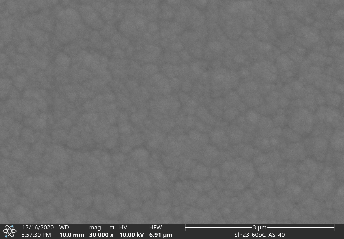


43.2 mA/cm^2^


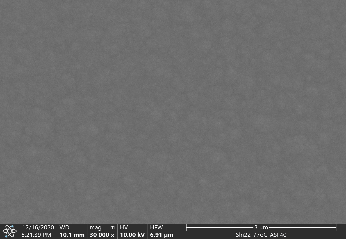


43.2 mA/cm^2^

**Figure S5**: SEM images of deposits at various solution temperature and current density at fixed 0.1 M Ni^2+^, 0.05 M MoO_4_^2-^, 0.25 M Cit^3-^, and 0.2 M NH_4_^+^. Scale bar: 3 µm.


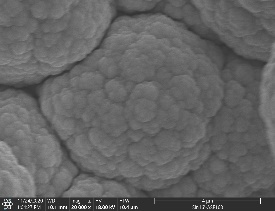


172.8 mA/cm^2^


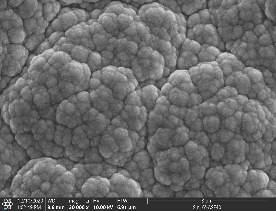


43.2 mA/cm^2^


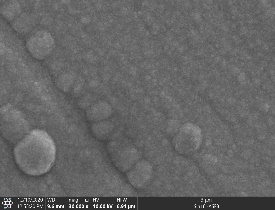


8.64 mA/cm^2^


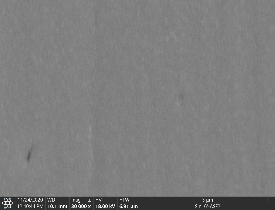


2.16 mA/cm^2^


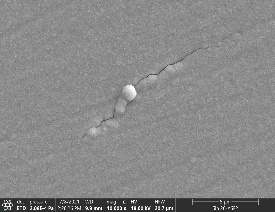


2.16 mA/cm^2^


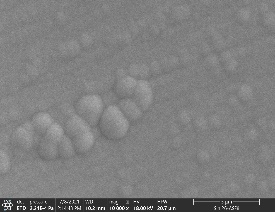


8.64 mA/cm^2^


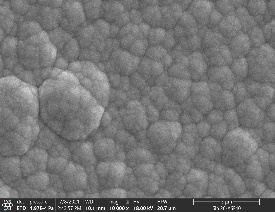


43.2 mA/cm^2^


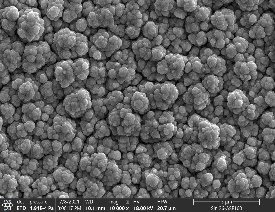


172.8 mA/cm^2^

No agitation

Agitation

**Figure S6**: SEM images of deposit as a function of agitation and current density at fixed 0.1 M Ni^2+^, 0.1 M MoO_4_^2-^, 0.05 M Cit^3-^ and 0.2 M NH_4_^+^. Scale bar: 4µm
